# Adaptive Response of Group B *Streptococcus* to High Glucose Conditions: New Insights on the CovRS Regulation Network

**DOI:** 10.1371/journal.pone.0061294

**Published:** 2013-04-09

**Authors:** Benedetta Di Palo, Valentina Rippa, Isabella Santi, Cecilia Brettoni, Alessandro Muzzi, Matteo Maria Emiliano Metruccio, Renata Grifantini, John L. Telford, Silvia Rossi Paccani, Marco Soriani

**Affiliations:** 1 Novartis Vaccines and Diagnostics, Siena, Italy; 2 Swiss Federal Institute of Technology, Lausanne, Switzerland; 3 Externautics S.p.a., Siena, Italy; University of Illinois at Chicago College of Medicine, United States of America

## Abstract

Although the contribution of carbohydrate catabolism to bacterial colonization and infection is well recognized, the transcriptional changes during these processes are still unknown. In this study, we have performed comparative global gene expression analysis of GBS in sugar-free *versus* high glucose milieu. The analysis revealed a differential expression of genes involved in metabolism, transport and host-pathogen interaction. Many of them appeared to be among the genes previously reported to be controlled by the CovRS two-component system. Indeed, the transcription profile of a Δ*covRS* strain grown in high-glucose conditions was profoundly affected. In particular, of the total genes described to be regulated by glucose, ∼27% were under CovRS control with a functional role in protein synthesis, transport, energy metabolism and regulation. Among the CovRS dependent genes, we found *bibA*, a recently characterized adhesin involved in bacterial serum resistance and here reported to be down-regulated by glucose. ChIP analysis revealed that in the presence of glucose, CovR binds *bibA* promoter *in vivo*, suggesting that CovR may act as a negative regulator or a repressor. We also demonstrated that, as for other target promoters, chemical phosphorylation of CovR in aspartic acid increases its affinity for the *bibA* promoter region. The data reported in this study contribute to the understanding of the molecular mechanisms modulating the adaptation of GBS to glucose.

## Introduction

*Streptococcus agalactiae* (GBS) is a Gram-positive β-haemolytic human pathogen commonly residing in the gastrointestinal tract of up to 50% of the healthy population. Although GBS is commonly associated with neonatal diseases [1] and postpartum infections, it is also an important cause of morbidity and mortality among adults [2]. GBS infections have been reported to occur in adults with serious underlying conditions [3], [4], [5], including HIV infections, liver cirrhosis and diabetes [6], [7]. For example, it has been reported that in diabetic patients GBS takes advantage of this condition by crossing the endothelial barrier and promoting invasion [8]. The impact of hyperglycemia upon susceptibility to GBS infection has not been fully elucidated, although, at least in part, this effect seems to be due to impairment of neutrophil effector functions [9]. This is supported by clinical evidence indicating a strong correlation between individuals with high blood glucose levels and the propensity to acquire GBS systemic infections [6], [7]. Nevertheless, carbohydrate catabolism has been highlighted to be important in the pathogenesis of streptococcal disease, with the number of mechanisms associated to the ability of streptococci to utilize both simple and complex sugars defined [10]. Of interest, a clear link between virulence factor production and complex carbohydrate catabolism in *Streptococcus pneumoniae*, *Streptococcus pyogenes* and GBS has been recently proposed by Shelburne and colleagues [11].

The ability of GBS to survive in specific human niches mainly depends on its capacity to activate a number of regulatory networks. This is achieved by controlling at a transcriptional level the production of proteins involved in adhesion, nutrient acquisition, and survival against host immune system [12], [13]. In particular, global gene expression analysis of GBS grown in amniotic fluid, blood and pH stress conditions [14], [15], [16] has recently revealed a number of mechanisms used by GBS to adapt to the host [13], [14], [15]. However the transcriptional network underlying the GBS response to glucose availability has been so far only marginally investigated.

Pathogenic streptococci use two-component regulatory systems (TCS) to sensing signals from the environment and efficiently respond to them. The capsule synthesis regulator (CovRS) is the most studied TCS in GBS, reported to be responsible for the modulation of transcription for up to 7% of total genes [12], [13]. The genes regulated by CovRS belong to different functional categories, such as cell envelope, cellular processes, metabolism and virulence factors.

In this study we setup an *in vitro* model to define the response of GBS to glucose. Comparative gene expression analysis revealed that not only many transport and metabolic genes were affected, but also genes involved in host-pathogen interactions. We also provide evidence that CovR controls approximately one third of glucose-dependent genes, including virulence determinants such as *bibA* and the *cyl* gene cluster. Moreover, we identified a conspicuous group of glucose-regulated genes independent from CovR control, whose regulation appears to involve other regulatory proteins. The data reported in this paper aim to improve the understanding of the physiological mechanisms underlying GBS adaptation to glucose-rich environments.

## Materials and Methods

### Bacterial strains and growth conditions

GBS type V strain 2603V/R and isogenic mutant strain 2603Δc*ovRS* come from an internal strain collection and have been previously described [12], [17]. *Escherichia coli* DH10BT1, HK100 and BL21 (DE3) were obtained from Invitrogen and used for cloning (DH10BT1, HK100) and expression (BL21 DE3) strains. Unless otherwise specified, for experiments testing the effect of glucose on transcriptional regulation, bacteria were grown in THB until late exponential phase, centrifuged and then resuspended in a complex medium (CM) containing 10 g/liter proteose peptone, 5 g/liter Trypticase peptone, 5 g/liter yeast extract, 2.5 g/liter KCl, 1 mM urea and 1 mM arginine. When they reached the mid exponential phase they were centrifuged, supernatant removed and resuspended in CM in the absence or presence of 55 mM glucose for 30 minute. *Escherichia coli* strains were grown in Luria–Bertani broth and supplemented with 100 µg ml^−1^ ampicillin.

### Microarray procedures and data analysis

Microarray comparison was performed for the wild-type strain 2603 V/R and the isogenic mutant strain Δ*covRS*, both grown as described in the previous paragraph. For each condition, four independent cultures were carried out in parallel. Total RNA was extracted from each culture with the RNeasy Mini Kit and treated with RNase-free DNase (both from Qiagen) according to the manufacturer's instructions. The concentration of total RNA was determined using a NanoDrop ND-1000 spectrophotometer (NanoDrop Technologies). RNA integrity was verified using a Bioanalyzer 1000 (Agilent). RNAs from each culturing condition were pooled and subjected to cDNA synthesis and labeling, which were performed at Roche NimbleGen, Inc. (Madison, WI, USA). Changes in gene expression levels were evaluated by using the NimbleGen GBS DNA microarray (17 probes for each gene, 3 replicates for probe consisting of 60-mer synthetic oligonucleotides for each gene). Hybridizations, staining, and processing were also performed at Roche NimbleGen. Scanned image and data were extracted and analyzed internally using NimbleScan software, as previously described [18]. Gene calls were generated using the Robust Multichip Average (RMA) algorithm as described in [19], [20]. The raw data were analyzed using the DNASTAR software. Genes whose expression ratios exceeded two fold at a P value of ≤0.01 were considered differentially expressed (T-Test with Bonferroni's correction). The microarray experiment has been submitted to the Array Express database of the European Bioinformatic Institute (http://www.ebi.ac.uk/microarray-as/ae/) and the submission accession number is E-MTAB-1100.

### Quantitative reverse transcriptase PCR

Reverse transcription-polymerase chain reaction (RT-PCR) was carried out on 1 µg total RNA using ImProm-II reverse transcriptase and random primers (Promega). Real-time quantitative PCR was performed using Fast start universal SYBR Green Master (Roche) according to manufactur's instruction, in a Light Cycler 480 System (Roche). All samples were run in triplicate on 96-well optical PCR plates (Roche Diagnostics). The specific primers used to amplify cDNA fragments corresponding to *potB*, *bibA*, *cfb*, *cylX*, *sap*, *sag1333*, *sag2021*, *sag0677* and *gyrA* are reported in Table S2. After an initial denaturation for 10 min at 95°C, denaturation at the subsequent 40 cycles was performed for 15 s at 95°C, followed by 15 s primer annealing at 60°C and a final extension at 72°C for 30 s. The ΔΔC_T_ method [21] was applied as a comparative quantification method. *potB*, *bibA*, *cfb*, *cylX*, *sap*, *sag1333*, *sag2021*, *sag0677* mRNA levels were normalized to *gyrA*, used as a housekeeping gene. The specificity of the amplified fragment was demonstrated by the melting curve, where a single peak was observed for each sample amplified with *potB*, *bibA*, *cfb*, *cylX*, *sap*, *sag1333*, *sag2021*, *sag0677* and *gyrA* primers.

### Cloning, production and purification of recombinant CovR

To produce a recombinant CovR as histidine–fusion protein in *E.coli*, *covR* DNA coding sequence (*sag1625*) was amplified by PCR from GBS type V strain 2603V/R. Oligonucleotides used for amplification of the covR gene incorporated an *Nde*I or *Xho*I site in the forward or reverse primers, respectively (Table S2), and were used to clone the gene into pET21b^+^ vector (Novagen). In order to express proteins, the plasmid containing *covR* gene was introduced into BL21 (DE3) (Invitrogen). *E. coli* BL21 (DE3) harboring *covR* was grown at 37°C to an OD_600_ of 0.5 in LB containing 100 µg ml^−1^ ampicillin. Expression of the recombinant protein was induced with 1mM isopropyl-beta-D-thiogalactopyranoside (IPTG, Sigma Aldrich) and the culture shaken for 3h at the same temperature. After 3 hours, the cells were harvested by centrifugation, resuspended and disrupted by sonication. The purification on the soluble fraction was performed with His Gravi Trap columns (GE Healthcare). Protein concentration was calculated using the Bradford assay [22]. After purification, the protein was dialyzed.

### Electrophoretic mobility shift assays (EMSA)

To test the binding of CovR to the *bibA* promoter, a biotinylated DNA fragment corresponding to the promoter region of *bibA* (*sag2063*) was amplified by PCR from the GBS type V strain 2603V/R (Table S2). Labeled probe was incubated with recombinant CovR for 20 minutes at room temperature. The reaction was stopped with 50% glycerol and protein-DNA complexes separated on native 6% polyacrylamide gel. DNA, transferred to a nylon membrane, was cross-linked to the membrane by UV light. After 1h incubation in blocking buffer, the membrane was then incubated with HRP conjugated streptavidin (Pierce) and signals visualized by chemiluminescence. Competitive EMSA experiments were performed to determine the specificity of CovR for the *bibA* promoter, by using an excess (250–500 fold) of either unlabelled *PbibA* (as a specific competitor) or the promoter region of *sag0017 (pscB)* gene (*Psag0017*, as a non-specific competitor) amplified by PCR from GBS type V strain 2603V/R chromosome using specific primers (*Fw sag0017* and *Rv sag0017*, Table S2). The *in vitro* phosphorylation of CovR was obtained incubating the recombinant protein with acetyl phosphate for 90 minutes at room temperature, as reported by Jiang *et al.* [23].

### Chromatin immunoprecipitation (ChIP)

2603 V/R GBS wild type strain and isogenic mutant strain Δ*CovRS* grown in CM in the presence or not of glucose were fixed with 1% formaldehyde and cross linking reaction was stopped by the addition of 125mM glycine. Bacteria were then sonicated reaching an average size of sheared DNA of ∼0.5 kb. Cell extracts were pre-cleared with protein A-Sepharose slurry (Pharmacia) for 45 minutes at 4°C and incubated overnight with an anti-CovR mouse serum. CovR-DNA complexes were immuno-precipitated with 50% protein A slurry for 3 hours at 4°C in sterile disposable mini-columns (Bio-Rad). After four washing steps, the immuno-complexes were treated with RNase A and digested overnight with proteinase K. Cross-linking was reversed for 6 hours at 65°C and DNA extracted with organic solvents. The presence of the target promoter sequences in the chromatin immunoprecipitates was detected by quantitative real-time PCR. Quantitative real-time polymerase chain reaction (qPCR) analyses were performed on a Light Cycler 480 System (Roche) using LightCycler 480 SYBR Green I Master mix (Roche). After an initial denaturation step for 10 min at 95°C, amplification was conducted for 50 cycles with 10 s at 95°C, followed by 10 s primer annealing at 50°C and final extension at 72°C for 20 s. The purified DNA from ChIP experiments before (input) and after (eluate) the immune-precipitation was used for qPCR. Primers sets for the amplification of *cylX*, *cfb*, *bibA* and *sag0017* promoter regions are listed in Table S2. The levels of each PCR product in the eluate was first normalized to input levels and then reported as fold changes with respect to wild type strain grown in CM without glucose.

## Results

### Regulation of GBS gene expression by glucose

**Figure 1:**
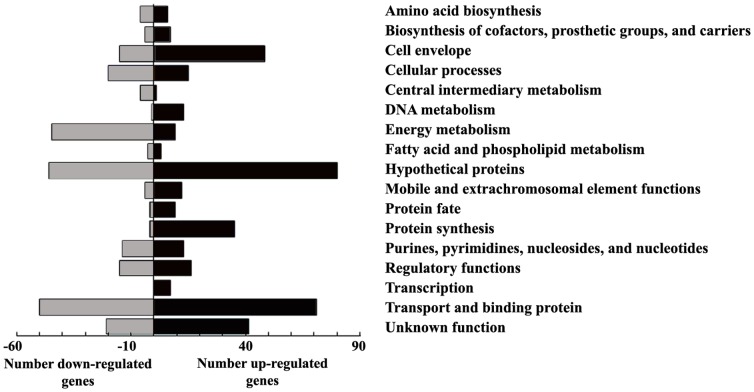
Differential regulation of gene expression in GBS strain 2603 V/R after exposure to 55 mM glucose. Genes were classified into 17 functional categories. Bars indicate the numbers of genes differentially regulated in medium with 55 mM glucose versus medium devoid of sugars.

To elucidate the response of GBS to glucose, we performed a comparative global gene expression analysis of the 2603 V/R GBS grown in THB until late exponential phase, centrifuged and then grown up to mid exponential phase in a peptone-based complex medium (CM) before been transferred for 30 minutes in a CM devoid of sugars (referred as no glucose condition) or containing 55 mM glucose (therein after referred as high glucose condition). A total of 640 genes were differentially regulated (>2-fold change, *P*<0.01), of which 257 were up-regulated and 383 were down-regulated. As expected, among the most regulated functional families, we found genes related to both energy metabolism and transport/binding (Fig.1). A list of the most regulated genes is reported in Table 1. The microarray data were validated by real-time RT-PCR analysis for eight genes (see Table S1).

**Table 1:** List of genes highly regulated in GBS strain 2603V/R after incubation in high glucose medium.

Family	TIGR locus	Annotation	Fold change	Regulation
**DNA metabolism**				
	*SAG0008*	Transcription-repair coupling factor *(mfd)*	6.1	Up
**Cell envelope**				
	*SAG0185*	Hypothetical protein	10.24	Down
	*SAG0281*	Hypothetical protein	7.04	Down
	*SAG2169*	Hypothetical protein	8.35	Up
**Unknown function**				
	*SAG0040*	ROK family protein	48.35	Down
	*SAG1643*	Glutamine amidotransferase, class I	19.91	Up
**Transport and binding proteins**				
	*SAG0034*	Sugar ABC transporter	128.03	Down
	*SAG1642*	ABC transporter	19.12	Up
	*SAG1949*	PTS system	84.29	Down
	*SAG1950*	PTS system	119.63	Down
	*SAG1951*	PTS system	92.69	Down
**Cellular processes**				
	*SAG0662*	CylX protein *(cylX)*	8.99	Down
	*SAG0663*	CylD protein *(cylD)*	11.63	Down
	*SAG0664*	CylG protein *(cylG)*	12.25	Down
	*SAG0666*	CylZ protein *(cylZ)*	9.92	Down
	*SAG0667*	CylA protein(*cylA*)	10.16	Down
	*SAG0668*	CylB protein *(cylB*	8.82	Down
	*SAG0669*	CylE protein *(cylE)*	8.79	Down
	*SAG0670*	CylF protein *(cylF)*	8.63	Down
	*SAG0671*	CylJ protein (*cylJ)*	7.01	Down
	*SAG0672*	CylI protein (*cylI)*	6.76	Down
	*SAG0673*	CylK protein (*cylK)*	5.98	Down
	*SAG1733*	Universal stress protein family	96.38	Up
	*SAG2043*	cAMP factor *(cfb)*	5.76	Down
**Transcription**				
	*SAG0777*	ATP- dependent RNA helicase	21.94	Up
**Energy metabolism**				
	*SAG0856*	Glycogen synthase *(glgA)*	20.31	Down
	*SAG1216*	Pullulanase *(sap)*	20.61	Down
**Amino acid biosynthesis**				
	*SAG1907*	Keto-hydroxyglutarate-aldolase (*eda-2)*	48.04	Down
	*SAG2165*	Ornithine carbamoyltransferase *(argF-2)*	10.84	Down

### Metabolic genes

The expression of genes involved in a wide range of metabolic pathways was found to be dramatically modulated after 30 minutes in 55 mM glucose, indicating a rapid adaptation of GBS central metabolism to the availability of new nutrients. Transcript changes were observed in genes involved in aminoacid biosynthesis, with *sag2165(argF-2)* and *sag2167(arc-2)*, encoding for a carbamate kinase and an ornithine carbamoyltransferase, being highly down-regulated (up to 20-fold). Both enzymes are components of the arginine deiminase system, which has been suggested to sustain bacterial survival in acidic environments by catalyzing the release of ammonia from arginine [24]. The *sag1907* gene (*eda-2*), encoding for keto-hydroxyglutarate-aldolase/keto-deoxy-phosphogluconate aldolase, was also found to be among the most down-regulated. Conversely, few genes were slightly up-regulated, such as the ones belonging to the aspartate and serine metabolism families. A drastic down-regulation (up to 130-fold) of genes implicated in the metabolism of complex sugars was observed, as well as the locus comprising the ORFs from *sag0033* to *sag0042*, codifying for components of the *nan* operon, responsible for the transport and metabolism of sialic acid. The list also encompasses: a carbohydrate kinase (*sag1906*) belonging to the PfkB family; a putative hexulose-6-phosphate synthase *sag1812(ulaD)*; L-a ribulose-5-phosphate 4-epimerase *sag1810(araD)*; a putative hexulose-6-phosphate isomerase (*sag1811*); and the *sag0118(rbsK)* gene encoding for a ribokinase. Genes encoding for proteins required for fermentation processes were also found to be down-regulated, including *sag1637*, *sag0053* and *sag0054*, annotated as alcohol dehydrogenases (*adh*, *adhP*, *adhE)*. The down-regulation of genes codifying for proteins implicated in the biosynthesis and degradation of polysaccharides such as *sag1901*, glucuronyl hydrolase; *sag0041*, acetyl xylan esterase; *sag1216*, pullulanase; *sag0856*, glycogen synthase (*glgA)*; *sag0854*, glucose-1-phosphate adenylyltransferase (*glgC)*; *sag0853* glycogen branching enzyme (*glgB)*, was also observed.

### Transport genes

Several genes encoding transport proteins were found to be up-regulated in high glucose conditions. These included *sag*0745, coding for a putative transporter of the NRAMP family, involved both in Mn^2+^ and Fe^2+^ uptake [25], [26] and genes encoding for proteins involved in potassium uptake, such as *sag1590*, *sag1591*, *sag1631* (belonging to the *trk* family) and *sag*1090. We also observed a fivefold up-regulation of the *sag1711* gene, coding for a putative CorA protein involved in magnesium transport [27]. Among the positively regulated genes, we found the *dps* gene *sag1444*, coding for a putative peptide/proton symporter [28]; a putative histidine ABC transporter (*sag0947* to *sag0949*); a spermidine-putrescine transporter, *potABCD* (*sag1108* to *sag1111*), implicated in the pathogenesis of *Streptococcus pneumoniae* [29]; the *sag0290* to *sag0292* operon (regulated up to 8-fold), encoding a putative polar amino acid ABC transporter; the *sag1145* gene, encoding for the sodium:alanin symporter protein; the *sag0715* to *sag0718* and *sag0947* to *sag0949* genes, encoding for amino acid ABC transporters. Furthermore, the transcription of genes encoding an ABC transporter specific for glycine betaine (*sag0241 to sag0244*), whose accumulation in *Bacillus subtilis* and *Lactococcus lactis* confers protection against osmotic and cold stress, was up-regulated [30], [31], [32]. As expected, in high glucose conditions, genes involved in the transport of complex carbohydrates were down-regulated, including: the region spanning from *sag1441* to *sag1443* ORFs, encoding for the maltose-maltodextrin transport system (*malE-F-G)*; *sag0955* and *sag1925* (*msmK*) genes, encoding a sugar-ABC transporter and a sugar transport ATP-binding protein, respectively; the ribose ABC transporter region (from *sag0114* to *sag0117*, namely *rbsA-B-C-D*); the cellobiose ABC transporter *sag0328(celC)* to sag0330(*celB).* Moreover several phosphotransferase systems (PTS), that allow the uptake of various carbohydrate sources, such as *sag1805*, *sag1813*, *sag1814*, *sag1948*–*1951*, *sag1898*–*1902* and *sag0192* were found to be highly down-regulated (up to 120-fold).

### Host-pathogen interaction genes

Pathogenic bacteria, by modulating the expression of surface-associated or secreted virulence factors, can adapt to host conditions and improve their capacity to persist in specific niches. In our experimental conditions the transcription of several known or putative virulence factors was down-regulated. In particular, the *cyl* gene cluster (*sag0662* to *sag0673*), required for GBS hemolysin production [33], and the *cfb* gene (*sag2043*), encoding the CAMP factor [34], were down-regulated in a range between 6 to12 fold. A similar pattern of expression was also observed for LPXTG cell wall-anchored proteins, such as BibA (*sag2063*). Among factors involved in host cell adherence and invasion and repressed by glucose, we report *sag1234*, a gene encoding for the laminin binding protein Lmb, which promotes GBS adherence to host cells through the binding to laminin [35]. Furthermore, we found that the expression of both *SodA* (*sag0788*), a gene which plays a crucial role in oxidative stress [36], and hyaluronate lysase (*sag1197*), which cleaves hyaluronic acid, a connective tissue component promoting GBS spreading during infection, was significantly reduced. On the contrary, *sag0677*, a gene encoding an unknown function LPXTG protein, and *sag2021*, codifying a GP-340 binding protein [37] were up-regulated by glucose. The same trend was observed for genes spanning from *sag1739(cydC)* to *sag1744*, grouped in an operon previously reported to be involved in the respiration metabolism and to play a role in virulence and GBS growth *in vivo* [38].

### Stress response genes

The incubation of GBS in a high glucose medium results in a considerable change in the expression of genes involved in adaptation and stress response. A dramatic change was observed in the transcript of *sag1677*, that codes for a universal stress protein, whose expression was highly down-regulated (27-fold) following exposure to glucose. Several genes, including *sag1135*, *sag1136* and *sag1137*, encoding for proteins involved in the stress response, were also down-regulated. Interestingly, these proteins are homologs to Gls24, a general stress protein of *Enterococcus faecalis* which has been reported to have a crucial role in stress response as well as in virulence [39].

### Transcriptional regulators

After 30 minutes of incubation with 55 mM glucose several transcriptional regulators were modulated. In particular, *sag1128*, *sag2017*, *sag0554*, belonging to the putative Cro/CI family and *sag1749*, *sag1655*, *sag0427*, being part of the putative Mer family of regulators, were 2–5 fold up-regulated. Of interest, the latter family has been reported to act as a key activator of the nitric oxide defense system in pneumococci, thus ensuring both survival and systemic infection [40], [41]. In response to the availability of glucose sources, bacteria down-regulate the expression of multiple genes involved in alternative sugar metabolism pathways through the carbon catabolite repression system (CCR), mediated by the catabolite control protein A (CcpA, *sag0707*). Bioinformatic analysis revealed that a number of transcriptional regulators, that were highly down-regulated under glucose conditions (up to 20-fold), has a *cre* box sequence in their promoter region (http://regprecise.lbl.gov/RegPrecise/gmregulon.jsp?gmproject_id=6875). They included: *sag0277*, encoding a Mga-like protein, a positive regulator of virulence in GAS [10]; *sag1348*, *fruR*, lactose phosphotrasferase system repressor; *sag0119*, *rbsR*, ribose operon repressor; *sag0042*, phosphosugar-binding transcriptional regulator belonging to RpiR family; *sag2073*, a transcriptional regulator belonging to GntR family and *sag2161*, encoding for a transcriptional regulator of the Crp/Fnr family.

### The response to glucose involves the two component system CovRS

**Figure 2:**
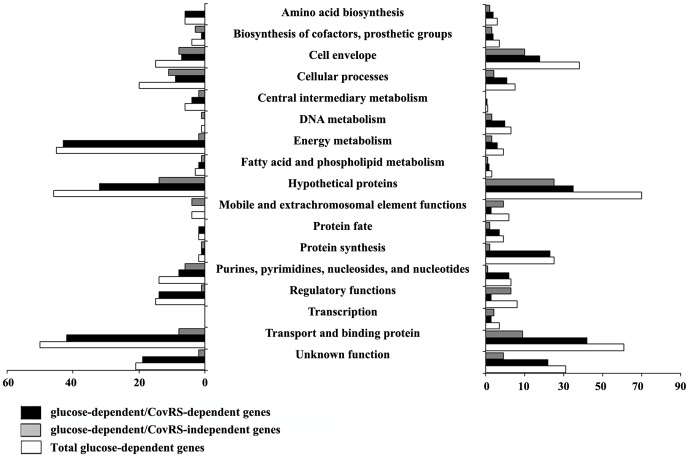
Transcriptome changes elicited by glucose in GBS. Comparison of gene expression changes (log2) between mid-log cultures of 2603 V/R wild-type strain and *CovRS* mutant following challenge with or without glucose. Data for the wild type and mutant strains are shown on the *x* axis and *y* axis, respectively.

**Figure 3:**
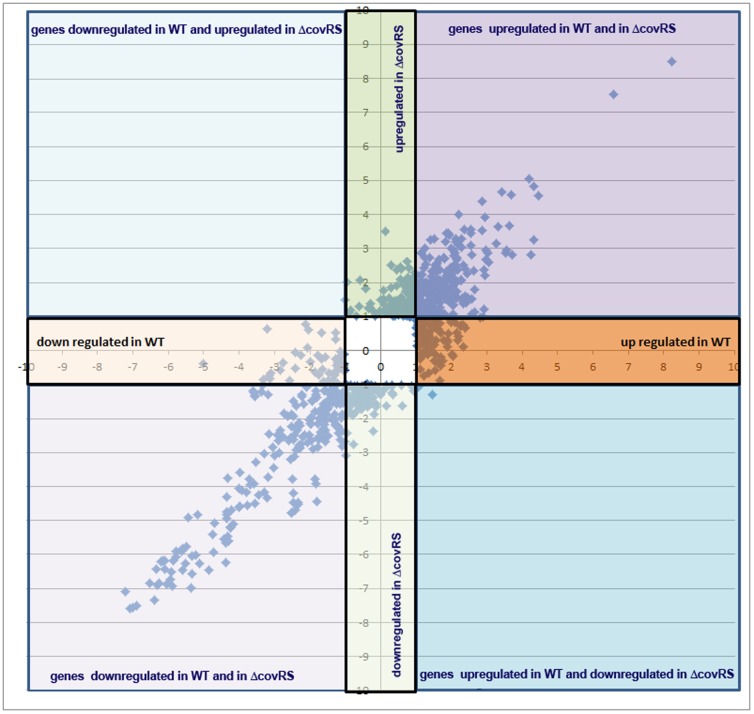
Differential regulation of gene expression in GBS strain 2603 V/R versus the isogenic Δ*CovRS* mutant strain after incubation in medium with 55 mM glucose versus a sugars-free complex medium. White bars indicate the number of glucose-regulated genes in the wild-type strain; black bars indicate the number of genes that are glucose- dependent and CovRS-dependent; grey bars indicate the number of genes that are glucose-dependent and CovRS-independent.

**Figure 4:**
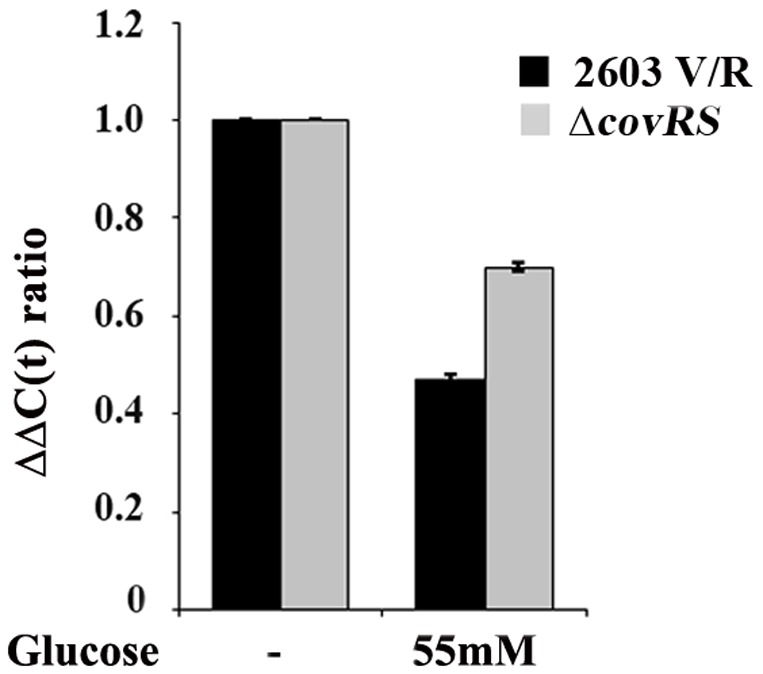
Real-time RT-PCR evaluation of *bibA* expression in 2603V/R and Δc*ovRS* strains grown in medium containing 55 mM glucose or in sugar-free medium. Transcript levels were normalized to the expression level of *gyrA*. Syber green runs were performed with cDNAs from the same reverse transcription reaction from 1 µg of total RNA. The ΔΔCT method was applied as a comparative method of quantification, using strains grown in sugar free medium as reference. The data are representative of 2 independent experiments, each in triplicate. Error bars, SD.

By comparing previously reported information on genes controlled by the CovRS two-component system [13] and the array of genes modulated by glucose, we found a number of them whose expression appeared to respond to both CovRS and glucose. We therefore investigated the transcriptional response of an isogenic Δ*covRS* mutant strain to glucose availability under the experimental conditions used for 2603 V/R GBS wild type (see Materials and Methods). Although WT and Δ*covRS* mutant strain growth rate during the experimental time span (30 min) was comparable in the absence or presence of glucose, at prolonged incubation time rate GBS growth rate was higher at 55mM glucose (Fig. S1). Of total genes differentially regulated under high glucose these genes, 466 (72.8%) were also regulated in the *covRS* mutant (defined as glucose-dependent-*covRS* independent genes) and showed the same regulation state of the wild type strain, while 174 genes were exclusively regulated in the wild type strain (referred as glucose dependent-*covRS* dependent genes), with 52 up-regulated and 127 down-regulated (Fig. 2). Finally, a group of 308 genes was only regulated by glucose in the *covRS* mutant, likely to involve different adaptive mechanisms compensating for the absence of a functional CovRS regulator. As shown in Fig. 3, the distribution of CovRS dependent and independent genes under high glucose conditions revealed important differences and a relevant role of CovRS in modulating genes involved in transport and binding of proteins, protein synthesis, energy metabolism and regulation. Conversely some functional classes appear to be independent from CovRS in presence of glucose, in particular genes up-regulated and having a role in regulation and transcription; genes both up and down-regulated and having a role in cell envelope biosynthesis and degradation. Glucose-CovRS dependent gene regulation was validated by real-time RT-PCR analysis for eight representative genes (Fig. 4) both in the wild-type and the *CovRS* mutant strains (Table S1).

### CovR binds bibA promoter in vivo

**Figure 5:**
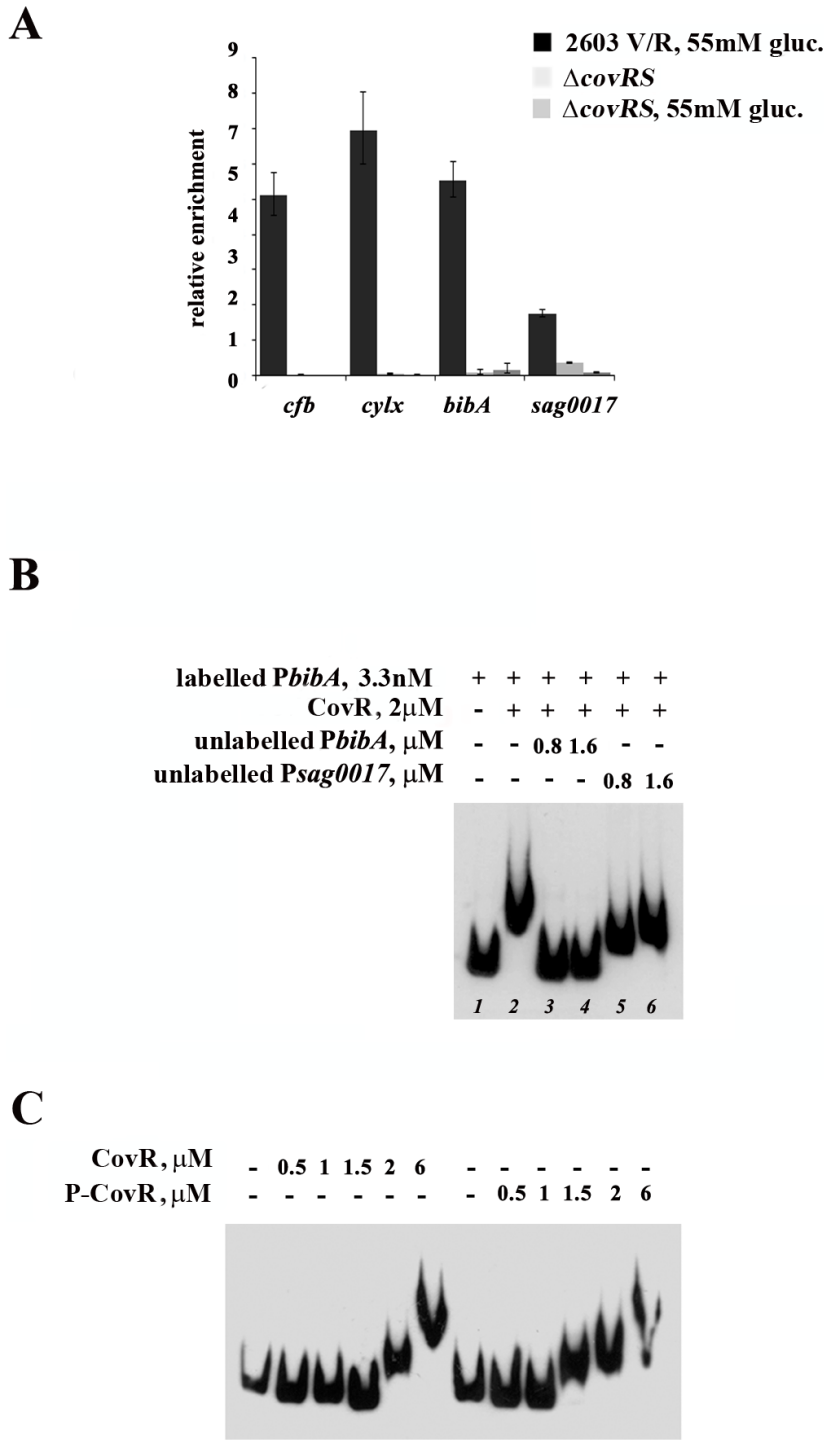
CovR binds to *bibA* promoter *in vivo*. (A) Quantification by qRT-PCR of *bibA* promoter immunoprecipitated with CovR antiserum in 2603 V/R wild type strain grown in medium devoid of glucose or in the presence of 55mM glucose. *cfb* promoter and *cylX* promoter were used as a positive control while *sag0017* promoter was used as a negative control. The level of PCR products of eluate from the isogenic Δc*ovRS* deletion mutant grown with or without glucose was negligible. The data are representative of 3 independent experiments, each in triplicate. Error bars, SD. (**B)** Competitive EMSA experiment. Labelled *PbibA* fragment (3.3 nM) was incubated without *(lane1)* or with CovR (2 µM) *(lane2*–*6)*, in the presence of different amounts of unlabelled *PbibA (lane 3*–*4)*, as a specific competitor, and *Psag0017 (lane5*–*6)*, as a non-specific competitor. The labelled DNA was detected by chemioluminescence. **(C)** CovR phosphorylation increases its affinity for *bibA* promoter. Electrophoretic mobility shift assay using recombinant CovR (left) and chemically phosphorylated recombinant CovR (right). Labelled *PbibA* DNA fragment (3.3 nM) was incubated without or with the indicated amounts of CovR. The labelled DNA was detected by chemioluminescence.

To better define the mechanism of action of CovRS on the transcription of putative virulence factors, we investigated the regulatory activity of CovRS on the promoter of *bibA*, an immunogenic bacterial adhesin with anti-phagocytic properties [42], here reported to be down-regulated by glucose in a CovRS dependent manner. The *bibA* promoter (*PbibA*) presents a specific binding motif for CovR, as reported by Lamy *et al.* [13]. We evaluated the binding of CovR to the *bibA* promoter *in vivo* by Chromatin immune-precipitation (ChIP) analysis of bacterial cultures exposed for 30 minutes to 55 mM glucose. Protein-DNA complexes were immunoprecipitated with CovR antiserum and analyzed by qRT-PCR for the presence of *bibA* promoter region, using *cylX* and *cfb* promoters as positive controls and *sag0017* promoter as negative. The amount of each promoter region in the eluate was first normalized to the amount in the input and subsequently reported as fold changes compare to wild type grown without glucose. As shown in Fig. 5A, quantification of the DNA immunoprecipitated with CovR antiserum revealed a 5.8 fold increase in the amount of *bibA* promoter region in GBS cultures exposed to high glucose (55 mM) levels compared to sugar-free conditions. This level is comparable to the levels of *cfb* promoter (4.7 fold) and *cylX* promoter (6.9 fold), whereas the negative control *sag0017* promoter shows little or no changes upon high glucose exposure. As expected in the eluate from the isogenic Δc*ovRS* deletion mutant grown with or without glucose the level of PCR products is close to the detection limit. These data clearly show that CovR in presence of high glucose is able to bind *bibA* promoter region *in vivo*.

### CovR affinity for PbibA depends on phosphorylation

To confirm that CovR interacts directly with the *bibA* promoter region, we performed electrophoresis mobility shift assay (EMSA) experiments by incubating a biotin-labeled DNA fragment corresponding to the promoter region of *bibA* with increasing amounts of recombinant CovR. We observed that the minimal quantity of protein necessary to induce a significant shift in the mobility of the *bibA* promoter was 2 µM (data not shown). To verify whether the binding of CovR to *PbibA* was specific, we performed competitive EMSA by incubating recombinant CovR protein with both 250- and 500-fold excess of unlabeled *PbibA* (specific competitor) or *sag0017*, a promoter lacking the binding motif for CovR (non-specific competitor). As displayed in Fig. 5B, the addition of the specific competitor abolished the binding of recombinant CovR to the labelled *PbibA* fragment, while the addition of increasing quantities of unlabeled *sag0017* had no effect on the ability of CovR to bind to *PbibA*. These data postulate a preferential binding of CovR to *PbibA*. Recently, Jiang and colleagues [23] demonstrated that chemical phosphorylation of CovR on aspartic acid, increased its affinity for the promoter of different genes. As shown in Fig. 5C, treatment of CovR with acetyl phosphate, a chemical phospho-donor, led to an increased affinity for the *bibA* promoter compared to the untreated recombinant CovR. The shift delay of the band relative to the phospho-CovR/*PbibA* complex was visible at a lower concentration than the one needed for the un-phosphorylated form of the protein. Altogether, these data confirm the capability of CovR to bind specifically the *bibA* promoter and that phosphorylation of CovR increases the binding to *PbibA*.

## Discussion

**Figure 6:**
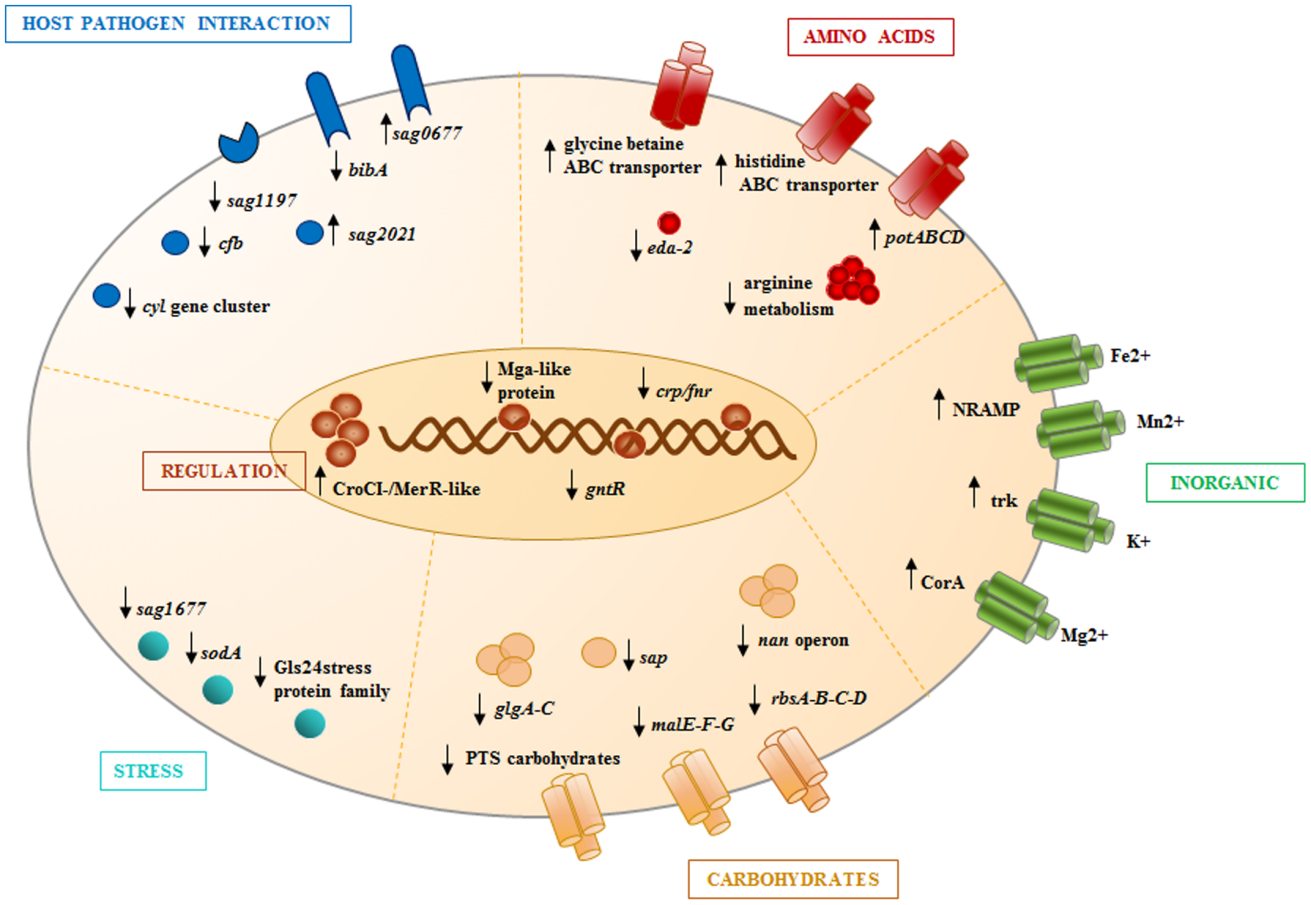
Graphical representation summarizing adaptive regulation of GBS in high glucose conditions. Genes of interest are color-grouped according to main functional categories. Arrows indicate up- or down-regulation relative to time of 30′ in high glucose *vs.* no glucose.

Glucose and other carbon sources are known to influence the lifestyle of many pathogens that adapt their metabolism to the nutritional composition of their host niches. Indeed, bacteria have evolved sophisticated methods for sensing carbon source availability to face the inter-bacterial competition for energy sources. For example, Alteri and co-workers have recently described a uropathogenic *Escherichia coli* subgroup that has adapted to grow as a harmless commensal in carbon-rich human intestine, but rapidly become invasive in the nutritionally poorer urinary tract [43]. Concerning *Staphylococcus aureus*, its colony spreading phenotype requires the expression of the *agr* gene, which is positively regulated by environmental glucose and carbon sources [44]. Our study proposes that a general trend of adaptive regulation is also triggered during the growth of GBS in high glucose conditions (Fig. 6). The *in vitro* model used in this study, although hardly resembling a physiological status of GBS, mimics the adaptation of this bacterium to standard laboratory conditions in which is common the use of high-glucose media (i.e. THB). One of the limitations for setting *in vitro* experiments looking at the effect of carbon sources on bacterial response is the fact that, differently from what happens *in vivo* where the system is rapidly buffered (i.e. blood) metabolites generated during the incubation of bacteria with sugars may alter the pH of the milieu. In order to precisely delineate the impact of glucose on global GBS transcriptome and avoid the contribution of pH alteration to the modulation of gene expression, we limited the duration of the experiments to 30 minutes, a time point at which pH remained unvaried (data not shown).

A number of reports have postulated that catabolism of carbohydrates plays a key role in the pathogenesis of invasive streptococci, including *Streptococccus pneumoniae*, *Streptococcus pyogenes* and GBS [10], [11], [45], [46]. Johns and colleagues have recently applied signature-tagged transport mutagenesis to a neonatal sepsis model and showed the importance of carbohydrate catabolism for GBS infectivity [46]. In particular, by knocking out genes involved in complex carbohydrate metabolism, including a maltose-binding protein (*mal*), a phosphotransferase (PTS) and a sucrose hydrolase (*scrB*), they observed an attenuated virulence [46]. These genes that appear to be essential to GBS infectivity *in vivo* were found to be down-regulated in our study, suggesting that the presence of high glucose levels may attenuate carbohydrate metabolism, rendering their expression dispensable. In line with this, we found a number of metabolic systems down-regulated by glucose such as genes involved in polyamine transport, biosynthesis and degradation of polysaccharides (including the *nan* operon, responsible for sialic acid catabolism) and fermentation processes. We also observed that bacteria grown in high glucose are dividing more rapidly (data not shown), thus, as expected, genes encoding for DNA replication, recombination, repair and for membrane biosynthesis were found to be highly up-regulated, while genes encoding for enzymes involved in substrate degradation were found to be down-regulated (see TableS3 and Table S4).

The exposure of GBS to glucose appears to modulate several genes coding for cell envelope proteins, likely to be involved in host-pathogen interactions. Although the number of up-regulated cell envelop genes is superior to the down-regulated ones, among the latter we found genes encoding for pore-forming toxins, such as hemolysin β, known to be crucial in promoting cell invasion and lysis [33], [34]. Furthermore, the presence of glucose also reduced the transcription of genes involved in host cell adherence, such as *Lmb* [35] and *bibA* [47]. On the contrary, *sag2021*, a GP-340-binding protein, proposed to be involved in bacterial colonization was found to be up-regulated [37]. The capacity of GBS to adapt to environmental changes mainly relies on two types of transcriptional regulation, the two-component gene regulatory systems (TCS) and stand-alone regulators (such as CcpA). The best characterized TCS in streptococci is the CovRS system, which plays a key role in connecting the expression of complex carbohydrate metabolism genes with that of virulence factors, thereby contributing to the modulation of the pathogenesis process [13], [48], [49]. In particular, the different CovRS mutants or alleles generate distinct signaling patterns and contribute to the bacterial ability to colonize specific host niches, disseminate and cause disease outcomes [50]. For instance, in Group A *Streptococcus*, pharyngeal and invasive strains show distinct virulence behaviors that are associated to fundamentally different transcriptomes designated as pharyngeal transcriptome profile (PTP) and invasive transcriptome profile (ITP). Interestingly, invasive strains show a mutated c*ovS* gene allele carrying 7-bp frameshift mutation. Experimental observation strongly support that the c*ovS* mutation is responsible for the PTP to ITP transition occurring during invasive infections [51]. Similarly, in GBS, there is a strain-specific regulatory role of CovRS that influences on the transition between commensal to virulent meningeal pathogen [52]. In our study, a wide range of glucose-dependent genes, in particular factors implicated in GBS transport function and host-pathogen interaction, resulted to be under CovRS regulation, strongly suggesting that effectors of this TCS are involved in the response and adaptation of GBS to high glucose condition. As previously reported for the response of GBS to pH stress conditions [16], also in the case of high glucose conditions the contribution of CovRS to the regulation of transcription of glucose-dependent genes was found to be independent on CovR abundance, as its transcription remained constant along the time span of the experiment. Our findings showing that CovR enhances its affinity for the *bibA* promoter in the presence of glucose by a phosphorylation dependent mechanism, postulate indeed that the mechanism of regulation is independent on CovR expression. Of interest, a few pH-dependent family genes reported by Santi and colleagues [14], [15], [16] were also found to be regulated by glucose-stress conditions. In particular genes involved in protein synthesis, transport and binding, cell envelop and several hypothetical proteins. This may indicate that GBS activates common pathways when exposed to shock conditions and similarly adapts to stress external stimuli such as low pH and high glucose.

Somewhat surprisingly, the analysis of the genes modulated by glucose in a CovRS defective strain revealed a consistent number of genes (∼ 300) regulated under such conditions but not in wild type bacteria. This phenotype may indicate that a number of alternative regulatory mechanisms are activated by glucose when CovR is not expressed. Indeed, we found that a dozen of transcriptional regulators, including the arginine repressor *argR* (*sag0500*) and members of the AraC family (*sag0432*) and Rgg/GadR/MutR family (*sag 1490*), were among the genes differently modulated by glucose in the Δ*CovRS* background. In addition, a number of genes encoding for DNA and RNA metabolism, amino acid transport and enzymes involved in substrate degradation were also found to be uniquely modulated by glucose in the CovRS mutant strain.

In this paper, by defining the response of GBS to glucose we observed an extensive re-modeling of bacterial transcriptome. We also provide evidence that under such conditions, CovR plays a pivotal role by controlling a large number of glucose-dependent genes. Future investigation are deserved to understand whether strain-specific CovRS mutations alter the adaptive response to glucose availability and how this reflects into the infectious lifestyle of this pathogen.

## Supporting Information

Figure S1 Growth curve of 2603V/R strain in medium containing 55mM glucose or in sugar-free medium. Bacteria were grown in THB at 37°C until late exponential phase, centrifuged and then resuspended in a complex medium (CM). When they reached the mid exponential phase they were centrifuged, supernatant removed and resuspended in CM in the absence or presence of 55 mM glucose.
(TIF)

Table S1 qRT PCR validation of regulated genes.
(XLS)

Table S2 Primers used in this study.
(XLS)

Table S3 Expression of glucose-dependent/CovRS-dependent genes.
(XLSX)

Table S4 Expression of glucose-dependent/CovRS-independent genes.
(XLSX)
