# Supplementary figures and images for: Adaptive Response of Group B Streptococcus to High Glucose Conditions: New Insights on the CovRS Regulation Network
Source: PLoS One. 2013 Apr 9;8(4):e61294. doi: 10.1371/journal.pone.0061294 (PMC3621830; doi:10.1371/journal.pone.0061294)

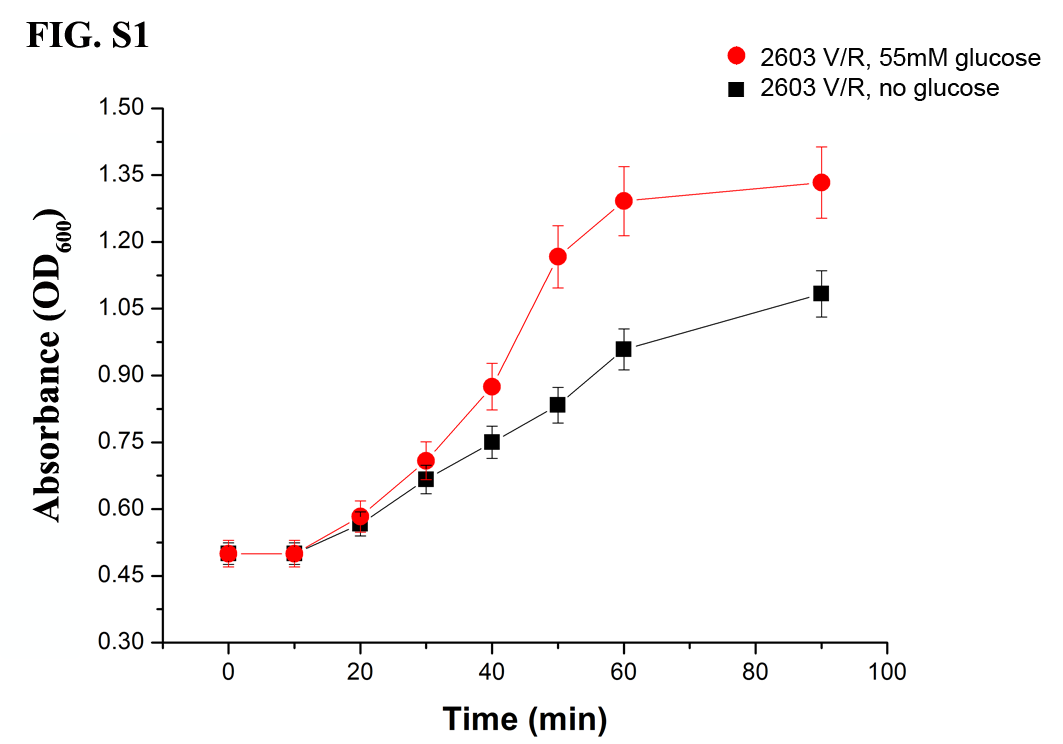

Supplement: Figure S1 — Growth curve of 2603V/R strain in medium containing 55mM glucose or in sugar-free medium. Bacteria were grown in THB at 37°C until late exponential phase, centrifuged and then resuspended in a complex medium (CM). When they reached the mid exponential phase they were centrifuged, supernatant removed and resuspended in CM in the absence or presence of 55 mM glucose. (TIF) [file pone.0061294.s001.tif]
